# Toll-like receptor 2 deficiency exacerbates corneal angiogenesis in injury by impairing regulatory T cells

**DOI:** 10.7150/thno.110322

**Published:** 2025-05-07

**Authors:** Jung Hwa Ko, Hyun Ju Lee, Hyeon Ji Kim, Jin Suk Ryu, Yoo Rim Choi, Chang Ho Yoon, Hyun Beom Song, Donghyun Kim, Ryang Hwa Lee, Joo Youn Oh

**Affiliations:** 1 Laboratory of Ocular Regenerative Medicine and Immunology, Seoul National University Hospital Biomedical Research Institute, Seoul, Korea; 2 Department of Ophthalmology, Seoul National University College of Medicine, Seoul, Korea; 3 Department of Tropical Medicine and Parasitology and Institute of Endemic Diseases, Seoul National University College of Medicine, Seoul, Korea; 4 Department of Biomedical Sciences, Seoul National University College of Medicine, Seoul, Korea; 5 Department of Microbiology and Immunology, Seoul National University College of Medicine, Seoul, Korea; 6 Department of Cell Biology and Genetics, Institute for Regenerative Medicine, Texas A&M University School of Medicine, College Station, TX, USA

**Keywords:** Angiogenesis, Cornea, Homeostasis, Monocyte, Regulatory T cell, Toll-like receptor 2, Vascular endothelial cell.

## Abstract

**Background:** Toll-like receptor (TLR) 2 is a primary sensor of injury, and regulatory T cells (Tregs) are crucial mediators of tissue homeostasis. In this study, we aimed to investigate whether TLR2 is necessary for Treg-mediated restoration of corneal homeostasis following injury.

**Methods:** We evaluated inflammatory corneal neovascularization and the proportions of Tregs, along with pro-angiogenic, pro-inflammatory monocytes, using a suture-induced corneal angiogenesis model in mice that either lacked TLR2 or were subjected to temporary TLR2 inhibition. The roles of injury-induced Tregs in corneal angiogenesis were further verified *in vivo* through adoptive transfer and *in vitro* using cultures of vascular endothelial cells.

**Results:** Inflammatory corneal neovascularization was significantly more pronounced in TLR2 knockout mice compared to wild-type mice, while no differences were observed in TLR4 knockout mice. Temporary TLR2 inhibition also exacerbated corneal neovascularization, whereas TLR4 inhibition did not. Mechanistically, corneal injury induced an increase in Tregs in wild-type mice, which was absent in TLR2 knockout mice. Conversely, pro-angiogenic, pro-inflammatory monocytes were elevated in TLR2 knockout mice. Adoptive transfer of injury-induced Tregs from wild-type to TLR2 knockout mice reduced corneal neovascularization and decreased the number of monocytes. Functional assays demonstrated that Tregs from TLR2 knockout mice exhibited lower cell proliferation and IL-10 secretion, but increased IFN-γ secretion compared to Tregs from wild-type mice. Furthermore, TLR2 knockout Tregs were less effective at inducing apoptosis and suppressing pro-inflammatory activation and tube formation of vascular endothelial cells than their wild-type counterparts.

**Conclusion:** Our findings suggest an expanded role for TLR2 in promoting corneal angiogenic and immunologic homeostasis during injury by regulating Treg numbers and functions.

## Introduction

Toll-like receptors (TLRs) are an evolutionarily-conserved family of molecules that recognize both pathogen-associated (PAMPs) and damage-associated molecular patterns (DAMPs) produced during infection or injury. Initially identified for their role in host defense against infection through detecting microbial components and triggering inflammatory responses 1, TLRs are also crucial for tissue protection and repair in sterile injury conditions, as they sense endogenous ligands released from dying or dead cells and mobilize cytoprotective responses 2. TLR2, in particular, is one of the most versatile TLRs, exhibiting affinity for a wide array of host-derived danger signals, including heat shock proteins, high mobility group box 1 protein, extracellular matrix fragments, and oxidation-associated carboxyethylpyrrole, as well as ligands from various pathogen sources, such as bacterial wall lipoproteins 3,4. Our group has previously identified TLR2 in the cornea as a primary sensor of the small heat shock protein HSPB4, a major DAMP released from injured keratocytes (corneal stromal fibroblasts) following sterile corneal injury 5.

As reflected by its versatility for ligands, TLR2 plays diverse roles in various biological processes, which largely depend on the cell or tissue type and the disease context. While TLR2 stimulation leads to classical pro-inflammatory activation 6, it can also evoke anti-inflammatory responses. For instance, polysaccharide A (PSA), an immunomodulatory molecule derived from the gut commensal *Bacteroides fragilis*, signals through TLR2 to induce Foxp3^+^ regulatory T cells (Tregs) and plasmacytoid dendritic cells, thereby promoting immune tolerance and offering protection against colitis 7-9. Furthermore, TLR2 on skin-resident cells is essential for the induction of myeloid-derived suppressor cells (MDSCs) and the suppression of T cell immunity following cutaneous infection with *Staphylococcus* aureus 10. *Listeria monocytogenes*, a facultative intracellular bacterial pathogen, also utilizes TLR2 to induce IL-10 secretion, thereby limiting pro-inflammatory cytokine secretion in bone marrow (BM)-derived macrophages and mice 11. Moreover, TLR2 expression in alveolar macrophages is upregulated by glucocorticoid treatment, and TLR2-containing extracellular vesicles from these macrophages inhibit inflammatory responses in vascular endothelial cells, contributing to glucocorticoid-induced immunosuppression 12. Interactions between hyaluronans and TLR2 have also been shown to provide signals that preserve epithelial cell survival and accelerate recovery from bleomycin-induced acute lung injury 13. Collectively, these findings suggest that TLR2 exerts both pro-inflammatory and anti-inflammatory functions to maintain tissue homeostasis, particularly at mucosal surfaces 14.

In addition to its roles in immunity, growing evidence underscore a key role for TLR2 in angiogenesis. Multiple studies have demonstrated that endothelial TLR2 enhances tumor- or hypoxia-driven angiogenesis 4,15. Conversely, other studies have shown that either blocking of TLR2 or genetic deficiency thereof increases neovascularization (NV) in a hindlimb ischemia model by inhibiting TLR2 on vascular endothelial cells or cKit^+^ BM stem cells 16,17. Furthermore, TLR2/4 deficiency in an oxygen-induced retinopathy model has been reported to prevent vascular degeneration and promote revascularization in the retina by downregulating Th17 cells and IL-17 18. These conflicting findings across studies reflect the complex and context-dependent functions of TLR2 in inflammation and angiogenesis. However, the precise mechanisms by which TLR2 exerts these effects remain unclear.

The cornea provides an ideal tissue microenvironment for investigating the role of TLR2 in immunologic and angiogenic homeostasis. Corneal transparency is essential for optimal optical function and vision. Thus, the cornea actively maintains avascularity, a phenomenon known as “corneal angiogenic privilege”. Excessive inflammation can disrupt this privilege, leading to inflammation-induced angiogenesis. Consequently, the cornea is equipped with multiple immune regulatory mechanisms, collectively referred to as "immunologic privilege". Notably, both corneal epithelial cells and stromal fibroblasts robustly express TLR2, and the cornea is enriched with TLR2 ligands during injury 5,19,20. Given these observations, we hypothesized that TLR2 plays a fundamental role in maintaining and restoring immunologic and angiogenic homeostasis in the cornea. To test this hypothesis, we investigated the functions and mechanisms of TLR2 using a model of sterile suture-induced inflammatory corneal NV.

## Methods

### Animal model and treatment

All procedures were conducted in accordance with the ARVO (Association for Research in Vision and Ophthalmology) statement on the use of animals in ophthalmic and vision research. The study was approved by the Institutional Animal Care and Use Committee of Seoul National University Hospital Biomedical Research Institute (Seoul, Korea).

TLR2 knockout (KO) mice and TLR4 KO mice, both with a C57BL/6 background, were purchased from the Jackson Laboratory (Bar Harbor, ME), and wild-type (WT) C57BL/6 mice were obtained from KOATECH (Pyeongtaek, Korea). All experiments utilized 8-week-old mice, including both males and females, and similar findings were observed for both sexes.

For the induction of corneal NV, mice were anesthetized via intraperitoneal (IP) injection of zolazepam-tiletamine (Zoletil^®^, Virbac, Carros, France) and topical administration of 0.5% proparacaine hydrochloride ophthalmic solution (Hanmi Pharm, Seoul, Korea). Three 10-0 nylon sutures were intrastromally placed in the cornea at 120˚ intervals, 2 mm from the center 21-23.

For TLR2 inhibition, either anti-TLR2 antibody (Ab) (Cat # mab2-mtlr2, InvivoGen, San Diego, CA) or control IgG (Cat # mabg1-ctrlm, InvivoGen) was intraperitoneally injected into WT C57BL/6 mice (10 μg per mouse) immediately following suture placement 16. For TLR4 inhibition, a TLR4 antagonist, LPS-RS (Ultrapure lipopolysaccharide from *Rhodobacter sphaeroides* Ultrapure, Cat # tlrl-prslps, InvivoGen) was administered both intraperitoneally (1 μg per mouse) and topically (50 μg/mL per mouse) to C57BL/6 mice immediately after the injury 24.

### Corneal NV measurement

Seven days after corneal suturing injury, corneal NV was quantified through clinical and histological examinations. We selected day 7 for the corneal NV analysis because this time point showed significant corneal hemangiogenesis and lymphangiogenesis as previously observed by our group and others 21,23.

For clinical assessment, the corneal surface was observed *in vivo* using a slit-lamp biomicroscope, and corneal NV was scored using a standardized scoring system as follows: the extent of corneal new vessels extending from the limbus toward the center was scored on a scale of 0 to 3 for each corneal quadrant. The overall corneal NV score was then calculated by summing the scores of the four quadrants (range 0 to 12) 21,24,25. Images of all eyes were captured using a digital camera mounted on the microscope.

For histological assessment, mice were humanely sacrificed, and their corneas were harvested for co-immunostaining of CD31 and LYVE-1. The excised corneas were fixed in a mixture of acetone and methanol (1:1) and incubated with anti-CD31 Ab (1:20) (Cat # 550274, BD Biosciences, Franklin Lakes, NJ) and anti-LYVE1 Ab (1:100) (Cat # 11-034, AngioBio, San Diego, CA). This was followed by incubation with goat anti-rat IgG-Cy3 (1:200) (Cat # A10522, Invitrogen, Waltham, MA) and goat anti-rabbit IgG-FITC (1:500) (Cat # A11034, Invitrogen). The stained tissues were flat-mounted onto slides and examined under fluorescence confocal microscopy (Olympus, Tokyo, Japan). Digital fluorescence photographs were analyzed using ImageJ software (National Institutes of Health, Bethesda, MD) as previously described 21-25. Briefly, after background subtraction and thresholding, the total area of the cornea was manually delineated by outlining the vessels of the limbal arcade as the border. The area stained for CD31 or LYVE-1 was measured by connecting the new vessel sprouts and calculating the area enclosed between this line and the limbal arcade. This value was then normalized as a ratio relative to the total corneal area.

### Quantitative reverse transcription polymerase chain reaction (qRT-PCR)

For qRT-PCR assays of the cornea, whole corneas were extracted from the eyeballs. For RNA extraction, corneal tissues and cells were incubated in RNA isolation reagent (RNA Bee, Tel-Test Inc., Friendswood, TX) and homogenized on ice using an ultrasound sonicator (Ultrasonic Processor, Cole Parmer Instruments, Vernon Hills, IL). Total RNA was isolated using the RNeasy^®^ Mini kit (Qiagen, Valencia, CA) and then converted to cDNA through reverse transcription with the High-Capacity RNA-to-cDNA^TM^ Kit (Applied Biosystems, Carlsbad, CA). Real-time PCR was performed on the ABI 7500 Real Time PCR System (Applied Biosystems) using specific TaqMan^®^ probes (TaqMan^®^ Gene Expression Assay kits, Applied Biosystems) and TaqMan^®^ Universal PCR Master Mix (all from Applied Biosystems). Data were normalized to *GAPDH* or *Gapdh* and analyzed using the 2^-∆∆Ct^ method, and results were presented as fold changes in mRNA levels relative to controls*.*

### Enzyme-linked immunosorbent assay (ELISA)

The cell-free supernatants were collected from cell cultures via centrifugation. The concentrations of IL-1β, IL-6, IL-10, IFN-γ, and CCL2 were measured using the DuoSet^®^ ELISA Kit (R&D Systems, Minneapolis, MN).

### Flow cytometry

Cells were isolated from spleens and ocular draining cervical lymph nodes (CLNs) by grinding the tissues between the frosted ends of two glass slides, filtering the homogenate through a 70-μm cell strainer (Corning Inc., Corning, NY), and centrifuging the resultant suspension. Blood cells were collected by centrifuging whole blood after treatment with ACK Lysing Buffer (Gibco, Waltham, MA). Single cell suspensions were stained with Abs against CD11b, Ly6C, Ly6G, CD4, CD25, and Foxp3 (all from eBioscience, Inc., San Diego, CA). For Foxp3 staining, cells were processed using the eBioscience™ Foxp3/Transcription Factor Staining Buffer Set (Invitrogen). The stained cells were analyzed using a flow cytometer (S1000EXi Flow Cytometer, Stratedigm, San Jose, CA), and the results were subsequently evaluated using FlowJo software (Tree Star, Inc., Ashland, OR).

### Cell sorting and adoptive transfer

CD11b^+^Ly6C^+^ cells were isolated from mouse spleens using fluorescence-activated cell sorting (FACS). Single-cell suspensions obtained from the spleens were stained with anti-CD11b and anti-Ly6C Abs (all from eBioscience, Inc.) in FACS buffer (Invitrogen). The stained CD11b^+^Ly6C^+^ cells were then sorted using the BD FACSAria™ III Cell Sorter (BD Biosciences).

Tregs were isolated from single-cell suspensions of mouse spleens using magnetic-activated cell sorting (MACS) with the CD4^+^CD25^+^ Regulatory T cell Isolation Kit (Miltenyi Biotec, Bergisch Gladbach, Germany) following the manufacturer's instructions. After isolation, the cells were analyzed by flow cytometry to confirm the purity of CD4^+^CD25^+^Foxp3^+^ Tregs, which was found to be 78.6 ± 3.3%.

Freshly-isolated CD11b^+^Ly6C^+^ cells were adoptively transferred into mice via tail vein injection (5 x 10^5^ cells in 100 μL Hank's balanced salt solution (HBSS) per mouse). Freshly-sorted Tregs were administered to mice either by IP injection (1 x 10^6^ cells in 100 μL HBSS per mouse) or by subconjunctival injection (1 x 10^5^ cells in 5 μL HBSS per mouse).

### Cell culture and stimulation

Primary keratocytes were isolated from the corneas of C57BL/6 WT or TLR2 KO mice using a previously-established protocol 26,27. Briefly, the corneal epithelium and endothelium-Descemet membrane were removed, and the corneal stroma was treated with 10 mg/mL dispase (Roche, Indianapolis, IN) at 37 ˚C for 90 min, followed by 200 U collagenase I (Worthington Biochemical Corp., Lakewood, NJ) for 30 min. The cells were collected by centrifugation and cultured in DMEM/F12 (1:1) (Welgene, Gyeongsan, Korea) containing 10% (vol/vol) fetal bovine serum (FBS) (Gibco) and 0.5% penicillin-streptomycin (PS) (Lonza, Valais, Switzerland). Passage 3 keratocytes were used for the experiments. To stimulate keratocytes, the cells were treated with 30 ng/mL recombinant mouse (rm) TNF-α (R&D Systems) and 1 ng/mL rmIL-1α (R&D systems) for 18 h.

The MACS-sorted Tregs were cultured in RPMI 1640 media (Welgene) supplemented with 10% (vol/vol) FBS (Gibco) and 1% PS (Lonza) in the presence or absence of 1 μg/mL anti-CD3 Ab (Invitrogen) and 25 IU/mL rmIL-2 (R&D Systems) for 5 d.

Passage 1 human umbilical vein endothelial cells (HUVECs) were obtained from Lonza (Cat # C2519A) and cultured in Endothelial Cell Growth Medium (EBM^TM^)-2 (Lonza). Passage 3 HUVECs were then stimulated with 10 U/mL recombinant human (rh) IFN-γ (R&D Systems) and 1 ng/mL rhTNF-α (R&D Systems).

In the HUVEC-Treg coculture studies, HUVECs were cocultured with either WT or TLR2 KO Tregs in anti-CD3 Ab/rmIL-2-coated transwell inserts (pore size 0.4 μm, MilliporeSigma, Burlington, MA) or in a direct coculture system at a HUVEC:Treg ratio of 3:1 under rhIFN-γ/rhTNF-α stimulation. The culture medium used was RPMI 1640 media (Welgene) containing 10% heat-inactivated FBS (Gibco).

### Treg proliferation assay

For evaluation of cell proliferation, Tregs were labelled with 5 μM carboxyfluorescein succinimidyl ester (CFSE) using the CellTrace™ CFSE Cell Proliferation Kit (Invitrogen) and then cultured for 5 d with or without anti-CD3 Ab/IL-2 stimulation. CFSE fluorescence intensity was analyzed by flow cytometry using the S1000EXi Flow Cytometer (Stratedigm).

### HUVEC apoptosis, tube formation, and migration assays

For the evaluation of cell apoptosis, HUVECs cocultured with either WT or TLR2 KO Tregs for 2 d were stained with Annexin V (ANX) and propidium iodide (PI) using the FITC Annexin V Apoptosis Detection Kit I (BD Biosciences). The percentage of ANX^+^PI^+^ cells was subsequently analyzed by flow cytometry using the S1000EXi Flow Cytometer (Stratedigm).

For the tube formation assay, HUVECs were seeded on Matrigel (Cat # 356237, Corning)-coated 24-well culture plates (6 x 10^5^ cells per well) and cocultured with either WT or TLR2 KO Tregs in anti-CD3 Ab/rmIL-2-coated transwells (2 x 10^5^ cells per well) under rhIFN-γ/rhTNF-α stimulation for 4 h. The degree of tube formation was quantified by counting the number of nodes of vascular tubes in randomly-selected fields at 100x magnification using an inverted microscope.

For the migration assay, HUVECs were seeded onto 24-well culture plates and allowed to stabilize for 24 h. Subsequently, portions of the HUVEC monolayer were excised using a scratcher and cocultured with either WT or TLR2 KO Tregs in transwells as previously described. The degree of cell migration was quantified by measuring the area of cells filling the gaps beyond a reference line using ImageJ software (National Institutes of Health) at 18 h post-scratch.

### Statistics

Statistical analysis was conducted using Prism software (GraphPad, San Diego, CA). Data are presented as mean ± SD. After assessing normality using the D'Agostino & Pearson test, Shapiro-Wilk test, or Kolmogorov-Smirnov test, the data were analyzed using one-way ANOVA with Tukey's test, the Kruskal-Wallis test with Dunn's multiple-comparisons test, or Student's* t*-test. Significant differences in means are indicated as follows: *p < 0.05, **p < 0.01, ***p < 0.001, and ****p < 0.0001.

## Results

### Genetic deficiency of TLR2, but not TLR4, exacerbates corneal NV after injury

**Figure 1:**
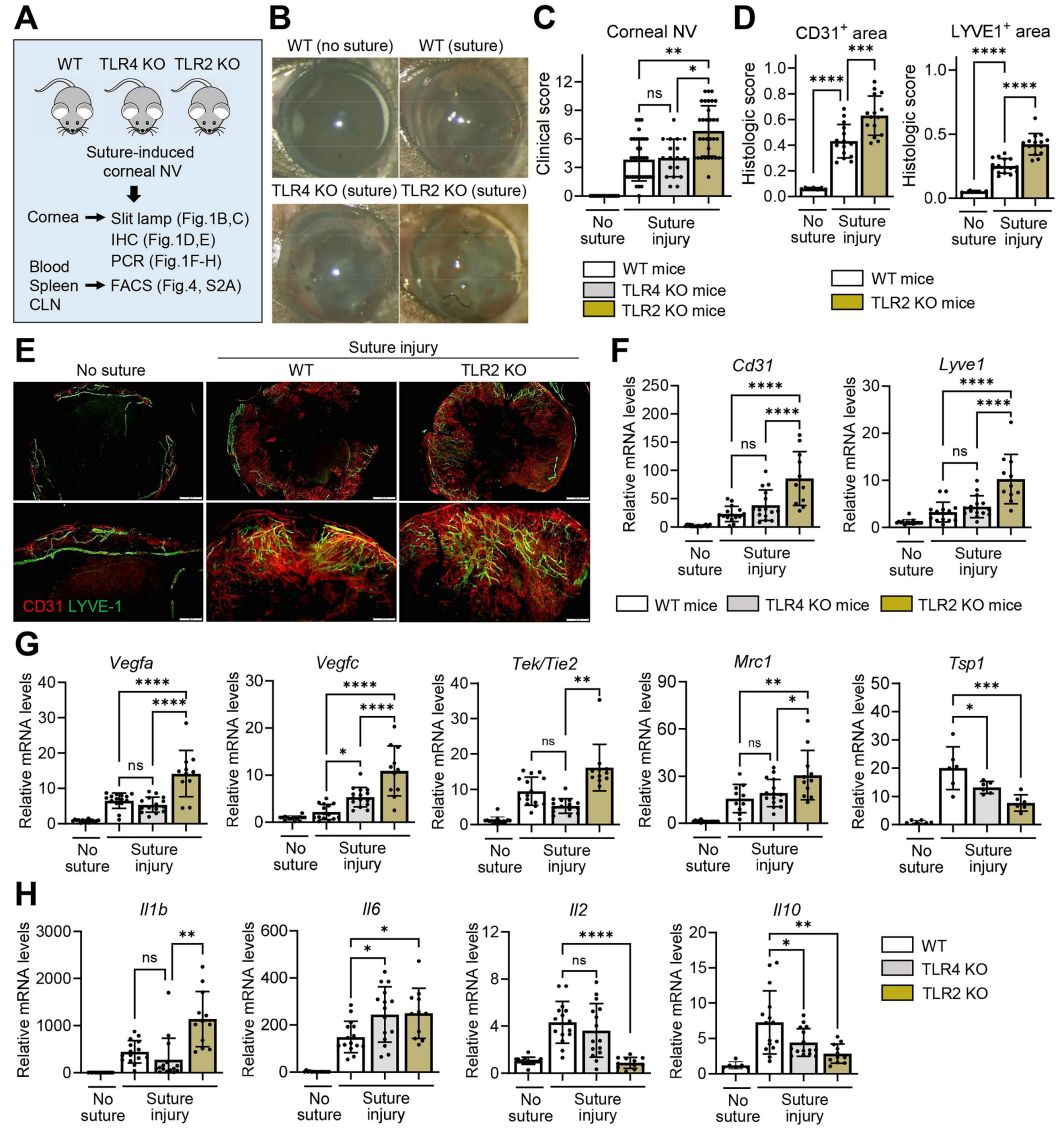
**Corneal NV is pronounced in TLR2 KO mice, but not in TLR4 KO mice, after sterile suturing injury. A.** Experimental scheme. TLR2 KO mice, TLR4 KO mice, and WT (C57BL/6) mice were subjected to 3 intrastromal corneal suture placements. The corneas, blood, spleens, and ocular draining cervical lymph nodes (CLN) were assayed 7 d post-injury. **B, C.** Representative slit-lamp biomicroscopic pictures showing corneal new vessels and opacity (B) and quantification of corneal NV as graded by the standardized clinical scoring system (C). n = 36 eyes for WT (suture) group, n = 32 eyes for TLR2 KO (suture) group, n = 18 eyes for TLR4 KO (suture) group, n = 15 eyes for WT (no suture) group. **D, E.** Quantitation of corneal NV as measured by CD31- and LYVE1-stained areas in corneal whole mounts (D) and representative microphotographs of corneal whole mounts with CD31 (red) and LYVE-1 (green) co-immunostaining (E). Scale bar: 500 μm (upper panel), 200 μm (lower panel). **F-H.** qRT-PCR assay of the cornea for endothelial cell markers (*Cd31* and *Lyve1*) (F), angiogenic markers (*Vegfa*, *Vegfc*,* Tek/Tie2,* and *Mrc1*) (G), and inflammatory cytokines (*Il1b*, *Il6*, *Il2,* and *Il10*) (H). mRNA levels are presented as fold changes relative to the levels in WT mice that did not receive any injury. Mean values ± SD are presented, where each dot represents the data from an individual mouse. Data are pooled from 3-5 independent experiments. *p < 0.05, **p < 0.01, ***p < 0.001, ****p < 0.0001, ns: not significant, as analyzed by one-way ANOVA with Tukey's test or by Kruskal-Wallis test with Dunn's multiple-comparisons test (C, *Tek/Tie2* in G, *Il1b* in H).

To examine the role of TLRs in the cornea during sterile injury, we induced corneal NV and inflammation by applying three 10-0 nylon sutures to the corneal stroma of TLR2 KO mice, TLR4 KO mice, and WT C57BL/6 mice using a sterile technique. We then compared the angiogenic and inflammatory responses (**Figure 1A**).

The development of corneal NV was significantly increased in TLR2 KO mice compared to TLR4 KO mice or WT mice as demonstrated by slit-lamp biomicroscopic observation of the corneal surface (**Figure 1B-C**), immunostaining of corneal whole-mounts with CD31 (a pan-endothelial marker) and LYVE-1 (a lymphatic vessel marker) (**Figure 1D-E**), and qRT-PCR assays for the levels of *Cd31* and *Lyve1* (**Figure 1F**). The levels of vascular growth factors, including *Vegfa* and *Vegfc*, and pro-angiogenic myeloid cell markers, such as *Tek/Tie2* and *Mrc1* 21, were significantly higher in the corneas of TLR2 KO mice compared to TLR4 KO or WT mice (**Figure 1G**). Conversely, the level of the anti-angiogenic factor *Tsp1* was lower in TLR2 KO mice (**Figure 1G**). Consistent with these angiogenic responses, the levels of pro-inflammatory cytokines *Il1b* and *Il6* were elevated in TLR2 KO mice compared to TLR4 KO or WT mice, whereas the levels of* Il2* and *Il10* were significantly reduced in TLR2 KO mice compared to WT mice (**Figure 1H**). However, there were no differences in corneal NV between TLR4 KO and WT mice (**Figure 1B-C, F**).

We also examined whether TLR2 deficiency affects the cornea under normal, uninjured conditions. The CD31/LYVE-1 co-immunostaining of the uninjured cornea indicated that limbal vasculature and corneal avascularity were maintained in TLR2 KO mice, similar to WT mice (**Figure S1A**). Furthermore, the corneal levels of angiogenesis-related molecules, including *Cd31*, *Lyve1*, *Vegfa*, *Vegfc*, *Tek/Tie2, Mrc1*, and *Tsp1,* showed no significant differences between TLR2 KO mice and WT controls (**Figure S1B-C**). Similarly, the levels of pro-inflammatory cytokines, such as *Tnfa*, *Il1b*, *Il12*, *Il18*, *Mmp9,* and *Il6*, did not differ between TLR2 KO mice and WT controls (**Figure S1D**). Interestingly, the levels of the chemokines *Cxcl8* and *Ccl2* were suppressed in TLR2 KO mice compared to WT controls, reflecting a decreased immunologic tone in TLR2-deficient corneas in the steady state (**Figure S1D**).

### Temporary TLR2 inhibition, but not TLR4 blocking, promotes corneal NV

**Figure 2:**
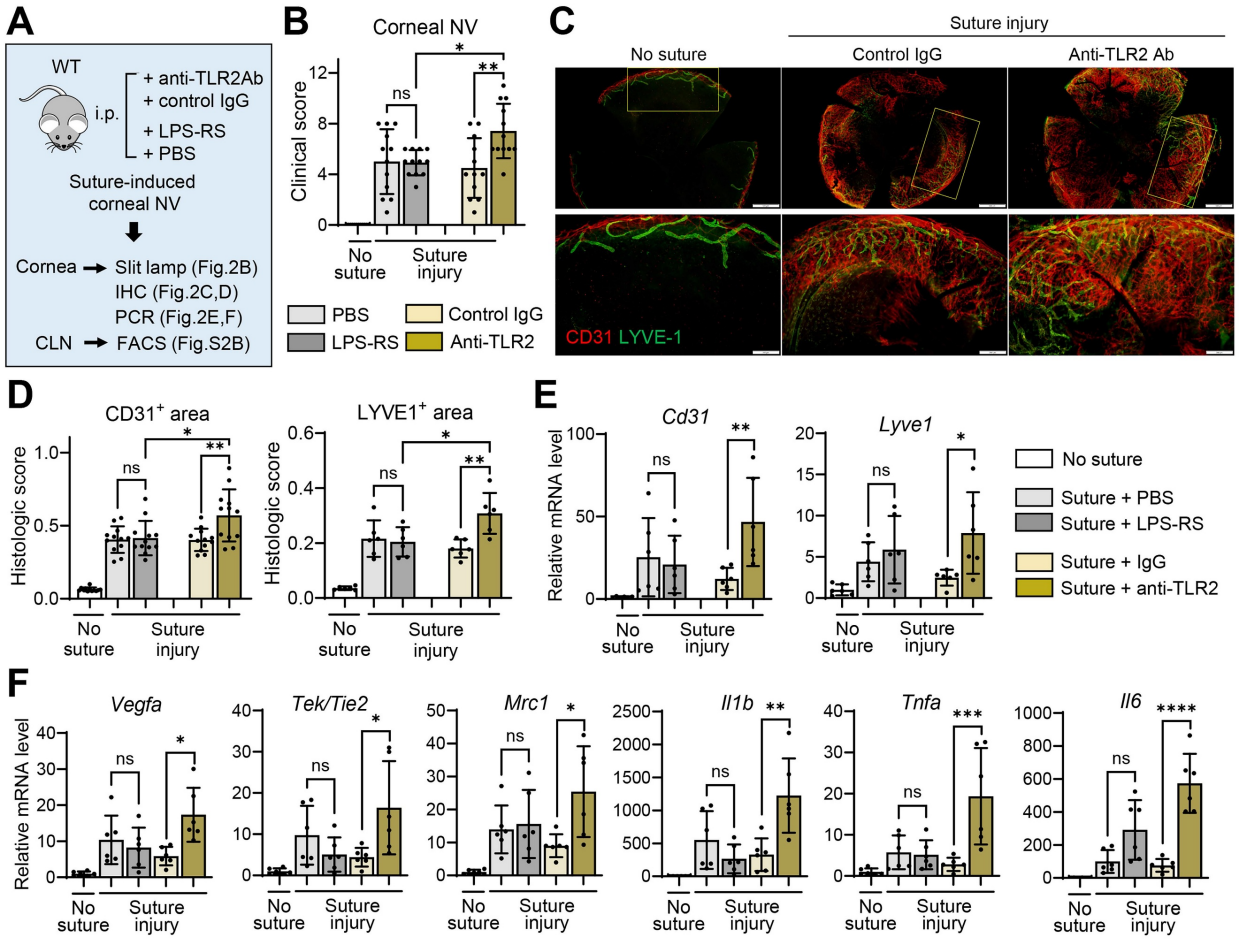
**Temporary TLR2 inhibition, but not TLR4 blocking, exacerbates corneal NV after sterile suturing injury. A.** Experimental design. WT (C57BL/6) mice were treated intraperitoneally (i.p.) with anti-TLR2 Ab, control IgG (control Ab for anti-TLR2 Ab), LPS-RS, or PBS (vehicle for LPS-RS) immediately after corneal suture placement. The corneas were evaluated 7 d after injury. **B.** Clinical scores of corneal NV graded under slit-lamp biomicroscopy. **C, D.** Representative microphotographs of CD31 and LYVE1 co-immunostaining of corneal whole mounts (C) and quantitation of CD31- and LYVE1-stained areas (D). Scale bar: 500 μm (upper panel), 200 μm (lower panel). **E, F.** qRT-PCR assays for angiogenesis-related markers and inflammatory cytokines in the cornea. mRNA levels are presented as fold changes relative to those in normal corneas without injury or treatment. Mean values ± SD are shown, where each dot depicts the data from an individual mouse. Data are pooled from 2-3 independent experiments. *p < 0.05, ** p < 0.01, ***p < 0.001, ****p < 0.0001, ns: not significant, as analyzed by one-way ANOVA with Tukey's test

Having observed that germline deletion of TLR2 promotes corneal NV following injury, we next determined whether temporary inhibition of TLR2 would produce similar outcomes. To block TLR2, we intraperitoneally injected either monoclonal anti-TLR2 Ab or control IgG into C57BL/6 mice immediately after corneal suturing injury (**Figure 2A**). For TLR4 inhibition, we administered either the TLR4 antagonist LPS-RS or phosphate-buffered saline (PBS, vehicle for LPS-RS) into C57BL/6 mice immediately after the injury (**Figure 2A**).

Both clinical and histological examinations showed more severe corneal NV development in mice treated with anti-TLR2 Ab compared to those treated with control IgG or LPS-RS (**Figure 2B-D**). In line with corneal NV, the levels of angiogenesis-related markers (*Cd31*, *Lyve1*, *Vegfa*, *Tek/Tie2,* and* Mrc1*) and pro-inflammatory cytokines (*Tnfa*, *Il1b,* and *Il6*) were significantly elevated in the corneas of anti-TLR2 Ab-treated mice relative to control IgG-treated mice (**Figure 2E-F**). In contrast, no significant differences in corneal NV or in the levels of angiogenic markers or pro-inflammatory cytokines were observed between mice treated with LPS-RS and those treated with PBS, indicating that TLR4 inhibition did not impact angiogenic or inflammatory responses following corneal injury (**Figure 2B, D-F**).

Collectively, these findings suggest that TLR2 plays a critical role in the regulation of pathological angiogenesis and inflammation in the cornea following sterile injury, while it is not involved in angiogenic or immunologic privilege under steady-state conditions. TLR4 does not exert such effects in either the injury or steady state.

### TLR2 deficiency does not induce immune activation in keratocytes

To gain mechanistic insights into the angiostatic and immunoregulatory actions of TLR2 in the cornea, we next examined the activation of local corneal cells and the systemic mobilization of immune cells in TLR2 KO mice compared with WT mice after corneal injury.

**Figure 3:**
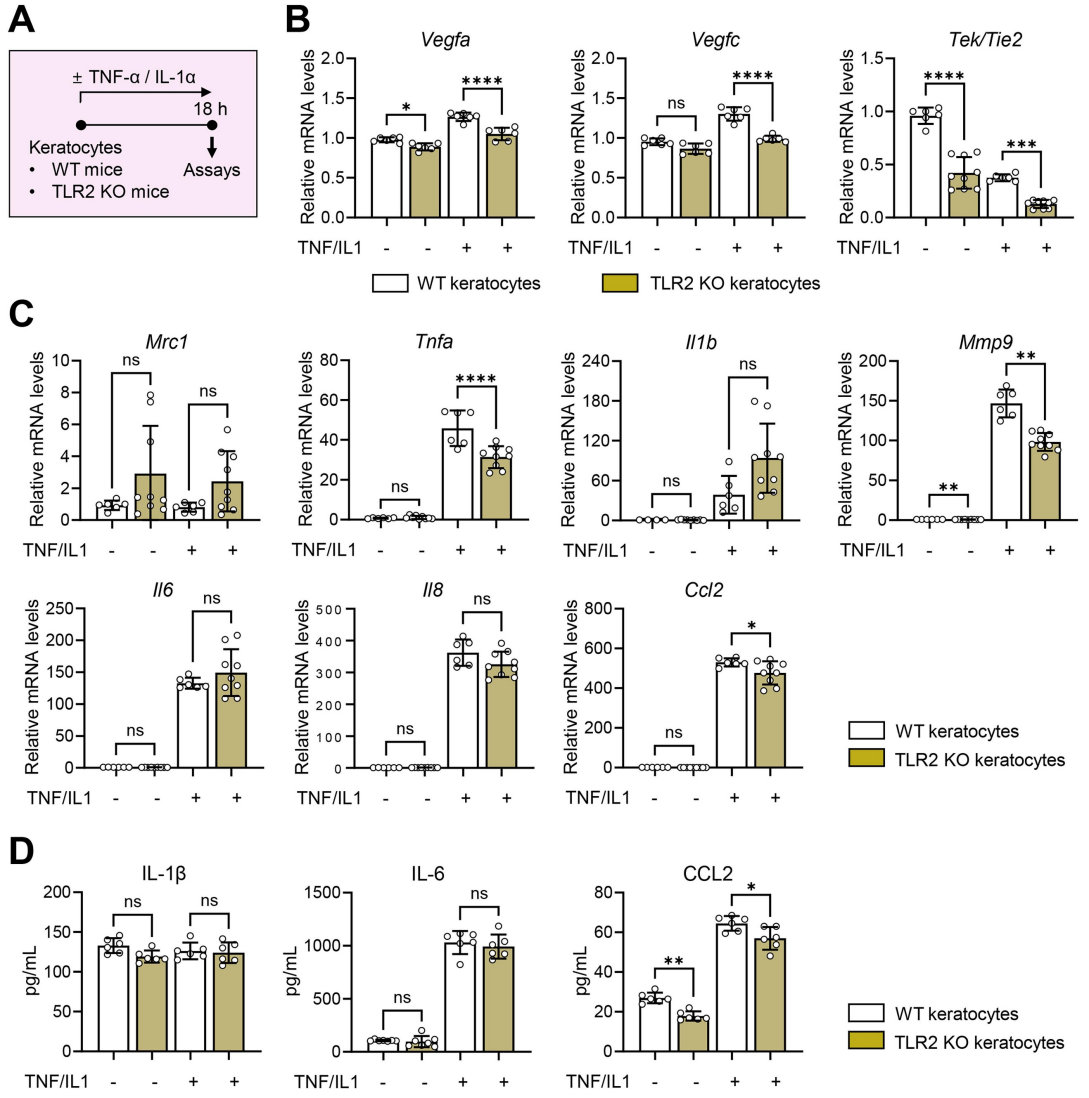
** Aberrant activation is not observed in response to inflammatory stimuli in TLR2 KO keratocytes compared to WT keratocytes.A.** Experimental protocol. Keratocytes were isolated from the corneas of WT (C57BL/6) mice and TLR2 KO mice and cultured for 18 h in the presence or absence of TNF-α and IL-1α. **B, C.** qRT-PCR assays of WT keratocytes and TLR2 KO keratocytes for angiogenesis-related factors and inflammatory cytokines/chemokines. mRNA levels are shown relative to those in WT keratocytes not treated with TNF-α or IL-1α. **D.** ELISA for secreted levels of inflammatory cytokines and chemokines in culture supernatants of WT keratocytes and TLR2 KO keratocytes. Mean values ± SD are presented. *p < 0.05, **p < 0.01, ***p < 0.001, ****p < 0.0001, ns: not significant, as analyzed by one-way ANOVA with Tukey's test or by Kruskal-Wallis test with Dunn's multiple-comparisons test (*Mrc1* and* Il1b* in C).

To evaluate the role of TLR2 in local corneal cells, we isolated keratocytes, the predominant resident stromal fibroblasts in the cornea, from TLR2 KO and WT mice. We then assessed the production of pro-angiogenic factors and pro-inflammatory cytokines/chemokines in keratocytes in the absence or presence of TNF-α and IL-1α which are major DAMPs released from dying cells that triggers acute inflammatory responses upon sterile tissue injury (**Figure 3A**) 28,29. The results showed that TLR2 KO keratocytes did not exhibit increased angiogenic or inflammatory responses compared to WT keratocytes, regardless of TNF-α/IL-1α stimulation. Specifically, the levels of *Vegfa, Vegfc, Tek/Tie2, Tnfa,* and *Mmp9* transcripts were lower in TNF-α/IL-1α-stimulated TLR2 KO keratocytes than in stimulated WT keratocytes (**Figure 3B-C**). Additionally, both transcript and secreted levels of CCL2 were decreased in TLR2 KO keratocytes compared to WT controls (**Figure 3C-D**), consistent with the observations from TLR2 KO vs. WT corneal tissues (**Figure S1D**). There were no significant differences in either transcript or secreted levels of IL-1β or IL-6 between TLR2 KO and WT keratocytes (**Figure 3C-D**).

These findings indicate that TLR2-deficient keratocytes do not display increased inflammatory activation compared to WT keratocytes, suggesting that the enhanced corneal angiogenic and inflammatory responses observed in TLR2-deficient mice may result from systemically-mobilized cell populations rather than local corneal cells.

### Foxp3^+^ Tregs are not induced, while monocytes increase, in TLR2 KO mice following injury

**Figure 4:**
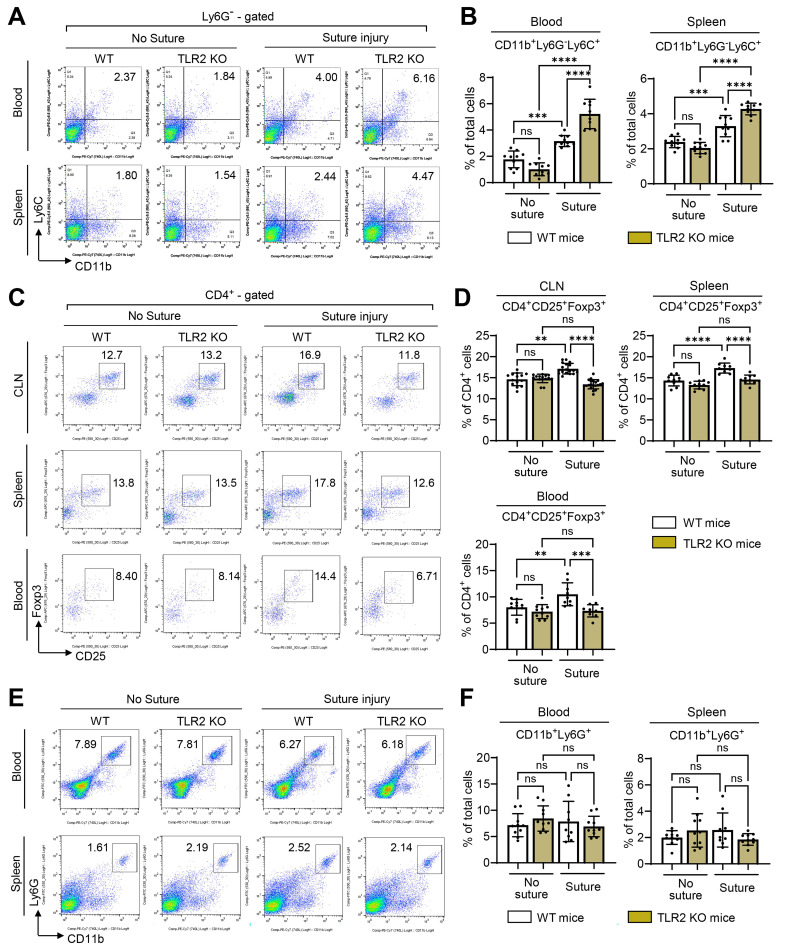
** Foxp3^+^ Tregs are not induced in TLR2 KO mice after injury, whereas monocytes are. A, B.** Representative (A) and quantitative flow cytometry results (B) for CD11b^+^Ly6G^-^Ly6C^+^ monocytes in the blood and spleen of WT (C57BL/6) mice and TLR2 KO mice without injury and 7 d after corneal suturing injury. **C, D.** Representative flow cytometry cytograms (C) and quantitation (D) of CD4^+^CD25^+^Foxp3^+^ Tregs in ocular draining cervical lymph nodes (CLN), blood, and spleen of WT mice and TLR2 KO mice without injury and 7 d post-injury. **E, F.** Representative and quantitative flow cytometry results for CD11b^+^Ly6G^+^ granulocytes in the blood and spleen of WT mice and TLR2 KO mice without injury and 7 d post-injury. Mean values ± SD are shown where each dot depicts the data from an individual mouse. Data are pooled from three independent experiments. **p < 0.01, ***p < 0.001, ****p < 0.0001, ns: not significant, as analyzed by one-way ANOVA with Tukey's test or by Kruskal-Wallis test with Dunn's multiple-comparisons test (CD4^+^CD25^+^Foxp3^+^ cells in CLN in D).

To identify the systemic cell populations affected by TLR2 deficiency and responsible for corneal angiogenesis, we examined monocytes and Tregs, both of which have been shown to contribute to corneal angiogenesis 21,30,31. Flow cytometric analysis revealed that corneal suturing injury caused significant increases in both CD11b^+^Ly6G^-^Ly6C^+^ monocytes and CD4^+^CD25^+^Foxp3^+^ Tregs in the blood, spleen, and CLNs of WT mice 7 d post-injury (**Figure 4A-D**). Notably, TLR2 KO mice exhibited a different response compared to WT mice. While CD11b^+^Ly6G^-^Ly6C^+^ monocytes were markedly elevated, CD4^+^CD25^+^Foxp3^+^ Tregs were not increased in response to injury (**Figure 4A-D**). Furthermore, the percentages of CD4^+^CD25^+^Foxp3^+^ Tregs in the blood, spleen, and CLNs of TLR2 KO mice 7 d post-injury were significantly lower than those in WT mice (**Figure 4C-D**). This decrease correlates with the reduced IL-10 levels observed in TLR2 KO mice after injury (**Figure 1H**), as Tregs are a primary source of IL-10. Conversely, the percentages of CD11b^+^Ly6G^-^Ly6C^+^ monocytes in the blood and spleen 7 d post-injury were significantly higher in TLR2 KO mice compared to WT mice (**Figure 4A-B**). Under normal conditions without injury, however, there were no significant differences in the proportions of CD4^+^CD25^+^Foxp3^+^ Tregs or CD11b^+^Ly6G^-^Ly6C^+^ monocytes between TLR2 KO and WT mice (**Figure 4A-D**).

Additionally, we assessed CD11b^+^Ly6G^+^ granulocytes. The analysis revealed that the percentages of CD11b^+^Ly6G^+^ granulocytes in the blood and spleen 7 d post-injury remained unaltered by either the injury or TLR2 deficiency (**Figure 4E-F**).

Furthermore, we evaluated TLR4 KO mice for CD11b^+^Ly6G^-^Ly6C^+^ monocytes, CD4^+^CD25^+^Foxp3^+^ Tregs, and CD11b^+^Ly6G^+^ granulocytes in the blood, spleen, and CLNs 7 d post-injury. No significant differences were found between TLR4 KO and WT mice in any of these cell populations (**Figure S2A**).

In agreement with the findings observed in TLR2 KO mice and TLR4 KO mice, TLR2 inhibition using anti-TLR2 Ab significantly reduced the percentage of CD4^+^CD25^+^Foxp3^+^ Tregs in CLNs of C57BL/6 mice after corneal injury, whereas TLR4 blocking with LPS-RS did not affect CD4^+^CD25^+^Foxp3^+^ Tregs (**Figure S2B**).

Taken together, these findings suggest that TLR2 deficiency results in the failure of CD4^+^CD25^+^Foxp3^+^ Treg induction and leads to the upregulation of CD11b^+^Ly6G^-^Ly6C^+^ monocytes following corneal injury, whereas TLR4 deficiency does not exert such effects.

### Increased Ly6C^+^ monocytes in TLR2 KO mice are pro-angiogenic

CD11b^+^Ly6C^+^ cells comprise two distinct cell populations with opposing functions: pro-inflammatory monocytes and anti-inflammatory MDSCs. Due to the lack of definitive markers, these two cell populations can only be distinguished by functional assays 32. Therefore, we proceeded to evaluate the functions of CD11b^+^Ly6C^+^ monocytes increased in TLR2 KO mice following corneal injury.

**Figure 5:**
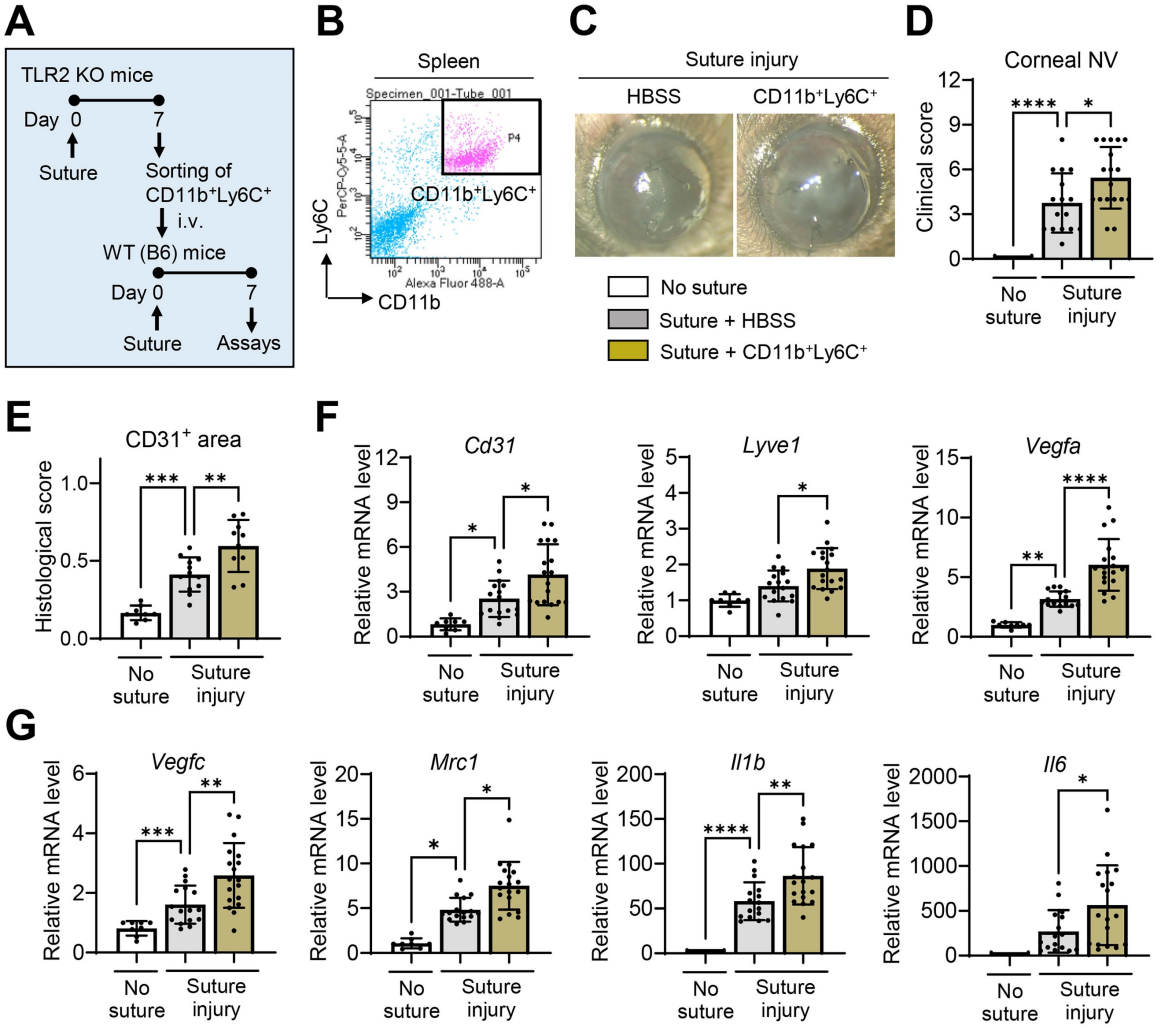
** Increased CD11b+Ly6C+ monocytes in TLR2 KO mice following injury promote corneal NV and inflammation.A.** Experimental design. CD11b^+^Ly6C^+^ monocytes were flow-sorted in the spleens of TLR2 KO mice 7 d after corneal suturing injury. Either the freshly-sorted CD11b^+^Ly6C^+^ monocytes or HBSS (Hank's balanced salt solution, vehicle for cells) were intravenously (i.v.) transferred into WT (C57BL/6) mice immediately after corneal suturing injury. The corneas and spleens were evaluated 7 d after the injury. **B.** Representative FACS plot schema showing the isolation of CD11b^+^Ly6C^+^ cells from the spleen of TLR2 KO mice 7 d post-injury. **C, D.** Slit-lamp biomicroscopic images of the cornea and clinical scores of corneal NV 7 d after injury and adoptive transfer of CD11b^+^Ly6C^+^ cells. **E.** CD31-stained area in corneal whole mounts. **F, G.** qRT-PCR assays for angiogenesis- and inflammation-related markers in the cornea. Shown are mRNA levels relative to the levels in normal corneas without injury or treatment. Mean values ± SD are shown, where each dot depicts the data from an individual mouse. *p < 0.05, **p < 0.01, ***p < 0.001, ****p < 0.0001, as analyzed by one-way ANOVA with Tukey's test, by Kruskal-Wallis test with Dunn's multiple-comparisons test (*Mrc1* in G) or by Student's* t*-test (*Il6* in G).

We isolated CD11b^+^Ly6C^+^ cells from the spleens of TLR2 KO mice 7 d after corneal suturing injury using FACS and intravenously transferred the freshly-isolated CD11b^+^Ly6C^+^ cells into WT mice immediately after the injury (**Figure 5A-B**). The results demonstrated that the adoptive transfer of CD11b^+^Ly6C^+^ monocytes significantly promoted corneal NV (**Figure 5C-E**). Additionally, the levels of pro-angiogenic markers (*Cd31, Lyve1, Vegfa, Vegfc,* and* Mrc1*) and pro-inflammatory cytokines (*Il1b* and* Il6*) in the corneas of mice that received the CD11b^+^Ly6C^+^ monocytes were elevated compared to those in HBSS-treated controls (**Figure 5F-G**). An increase in splenic size was also observed in these mice after the transfer of CD11b^+^Ly6C^+^ monocyte (**Figure S3**).

Thus, the results indicate that CD11b^+^Ly6C^+^ monocytes, which are elevated in TLR2 KO mice following corneal injury, exhibit both pro-angiogenic and pro-inflammatory characteristics.

### Foxp3^+^ Tregs attenuate corneal NV and reduce monocytes

In the aforementioned experiments, we observed that TLR2 deficiency led to impaired induction of Tregs following corneal injury, which was accompanied by an increase in pro-angiogenic, pro-inflammatory monocytes. Based on these observations, we hypothesized that Tregs suppress pro-angiogenic, pro-inflammatory monocytes and mitigate pathological corneal angiogenesis.

**Figure 6:**
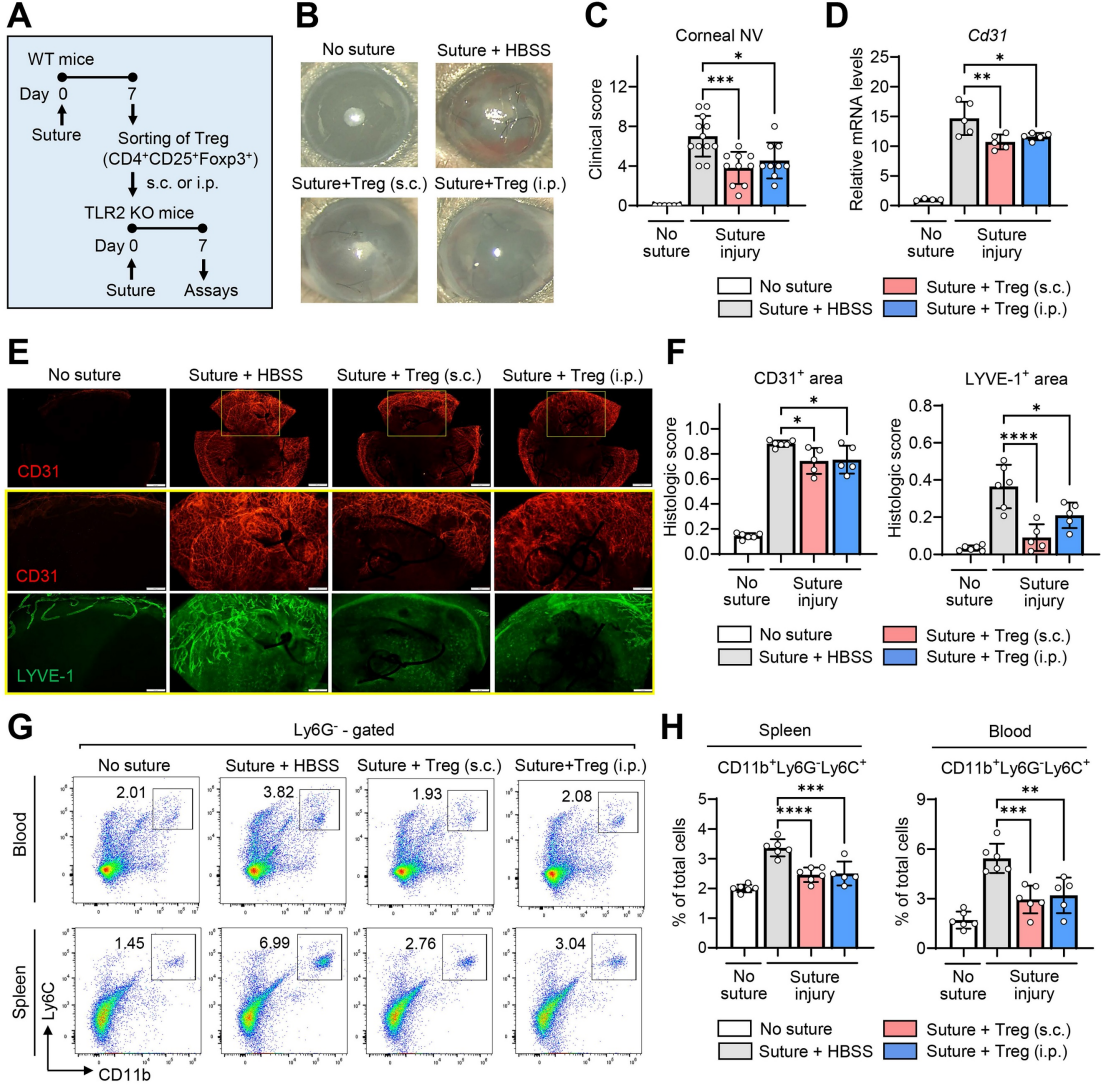
** Tregs induced by corneal injury reduce corneal NV and CD11b+Ly6C+ monocytes. A.** Experimental scheme. CD4^+^CD25^+^Foxp3^+^ Tregs were sorted from the spleens of WT (C57BL/6) mice 7 d after corneal suturing injury. The isolated cells were transferred into TLR2 KO mice via either subconjunctival (s.c.) or intraperitoneal (i.p.) injection immediately after injury, and 7 days later, the cornea, spleen, and blood were assayed. **B, C.** Slit-lamp biomicroscopic images of the cornea (B) and clinical scores of corneal NV (C). **D.** qRT-PCR assay for pan-endothelial marker *Cd31* in the cornea. mRNA levels are presented as fold changes relative to the levels in the cornea of TLR2 KO mice that did not receive injury or treatment. **E.** Double CD31 (red) and LYVE-1 (green) immunostaining of corneal whole mounts. Scale bar: 500 μm (upper panel), 200 μm (lower two panels). **F.** Quantitation of CD31- and LYVE-stained areas in corneal whole-mounts. **G, H.** Flow cytometric analysis of the spleen and blood for CD11b^+^Ly6G^-^Ly6C^+^ monocytes. Mean values ± SD are presented, where each dot represents the data from an individual mouse. *p < 0.05, **p < 0.01, ***p < 0.001, ****p < 0.0001, as analyzed by one-way ANOVA with Tukey's test

To test this hypothesis, we purified CD4^+^CD25^+^Foxp3^+^ Tregs from the spleens of WT mice using MACS microbeads 7 d after suturing injury to the cornea (**Figure S4**), and transferred the freshly-purified Tregs into TLR2 KO mice via either subconjunctival or IP injection immediately following corneal injury (**Figure 6A**).

Both subconjunctival and IP administration of CD4^+^CD25^+^Foxp3^+^ Tregs markedly alleviated the development of corneal NV in TLR2 KO mice as assessed by clinical, histological, and molecular examinations (**Figure 6B-F**). Furthermore, the adoptive transfer of CD4^+^CD25^+^Foxp3^+^ Tregs significantly reduced the percentages of CD11b^+^Ly6G^-^Ly6C^+^ monocytes in the blood and spleen of TLR2 KO mice (**Figure 6G-H**).

These results demonstrate that CD4^+^CD25^+^Foxp3^+^ Tregs induced by corneal injury suppress pro-angiogenic monocytes and inhibit the progression of corneal NV, indicating a homeostatic mechanism for the regulation of pathologic corneal angiogenesis under sterile injury. Importantly, these Tregs are not increased in TLR2-deficient mice after injury, pointing to the crucial role of TLR2 in the activation of the negative feedback loop that involves Tregs and pro-angiogenic monocytes for the homeostatic regulation of corneal angiogenesis.

### TLR2 KO Tregs exhibit lower cell proliferation and IL-10 secretion

**Figure 7:**
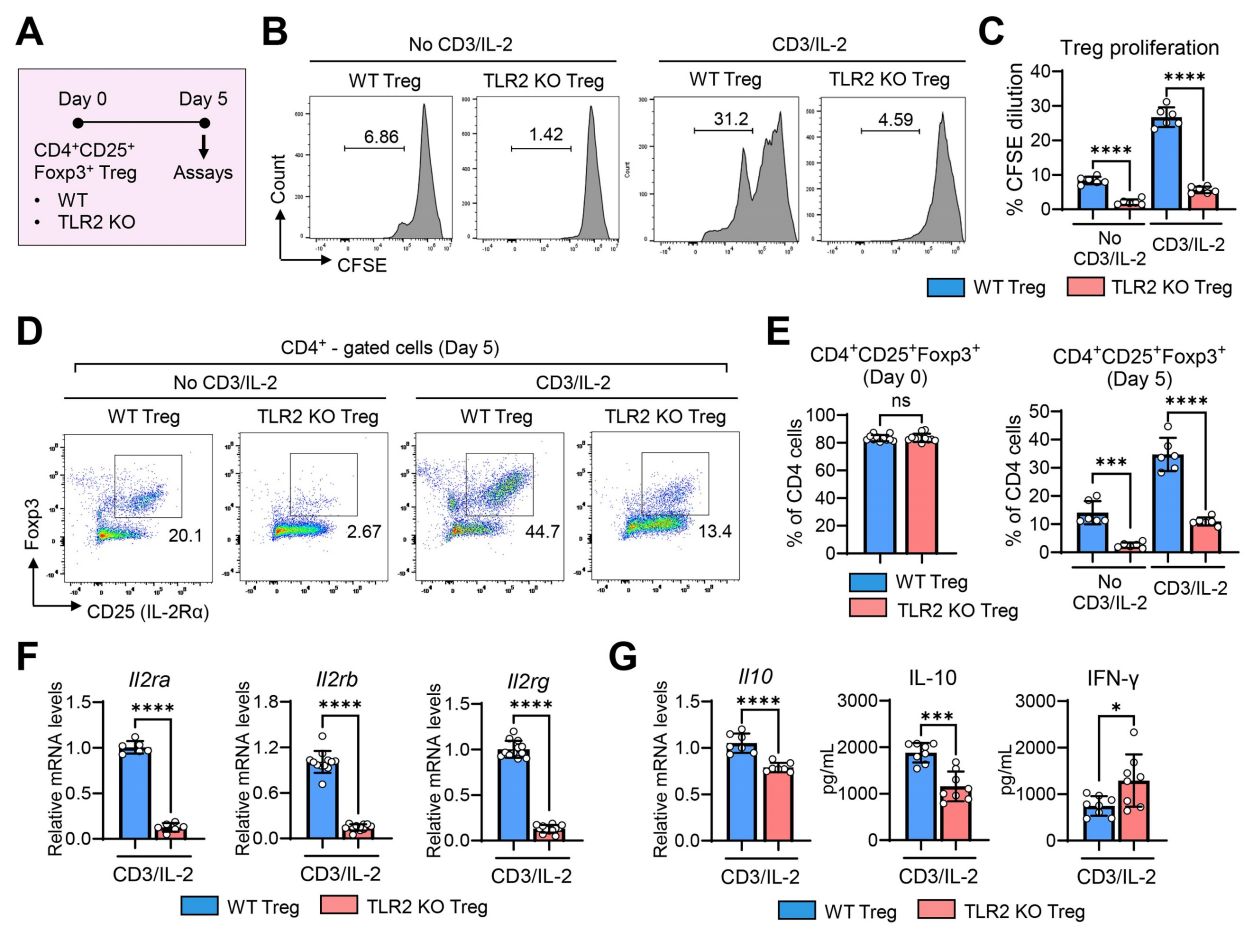
** TLR2 KO Tregs exhibit reduced cell proliferation and IL-2 receptor expression, lower IL-10 secretion, and higher IFN-γ secretion. A.** Experimental scheme for comparative analysis of WT (C57BL/6) Tregs and TLR2 KO Tregs. CD4^+^CD25^+^Foxp3^+^ Tregs were isolated from the spleens of WT mice and TLR2 KO mice and cultured for 5 d in the presence or absence of anti-CD3 Ab and IL-2. Then, assays were performed for evaluation of cell proliferation, marker expression, and cytokine secretion. **B, C.** Representative flow cytometry histograms of CFSE dilution assay (B) and quantitation of cell proliferation (C). **D, E.** Representative (D) and quantitative flow cytometric analysis (E) for expression of intrinsic Treg markers CD25 and Foxp3 in WT Tregs and TLR2 KO Tregs over time in culture. The percentage of CD4^+^CD25^+^Foxp3^+^ cells out of CD4^+^ cells is presented. **F.** qRT-PCR for transcript levels of *Il2ra*, *Il2rb*, and *Il2rg*. **G.** qRT-PCR and ELISA for transcript and secreted levels of IL-10 and IFN-γ. Mean values ± SD are presented. *p < 0.05, ***p < 0.001, ****p < 0.0001, ns: not significant, as analyzed by one-way ANOVA with Tukey's test or Student's* t*-test (CD4^+^CD25^+^Foxp3^+^ (Day 0) in E, F and G).

**Figure 8:**
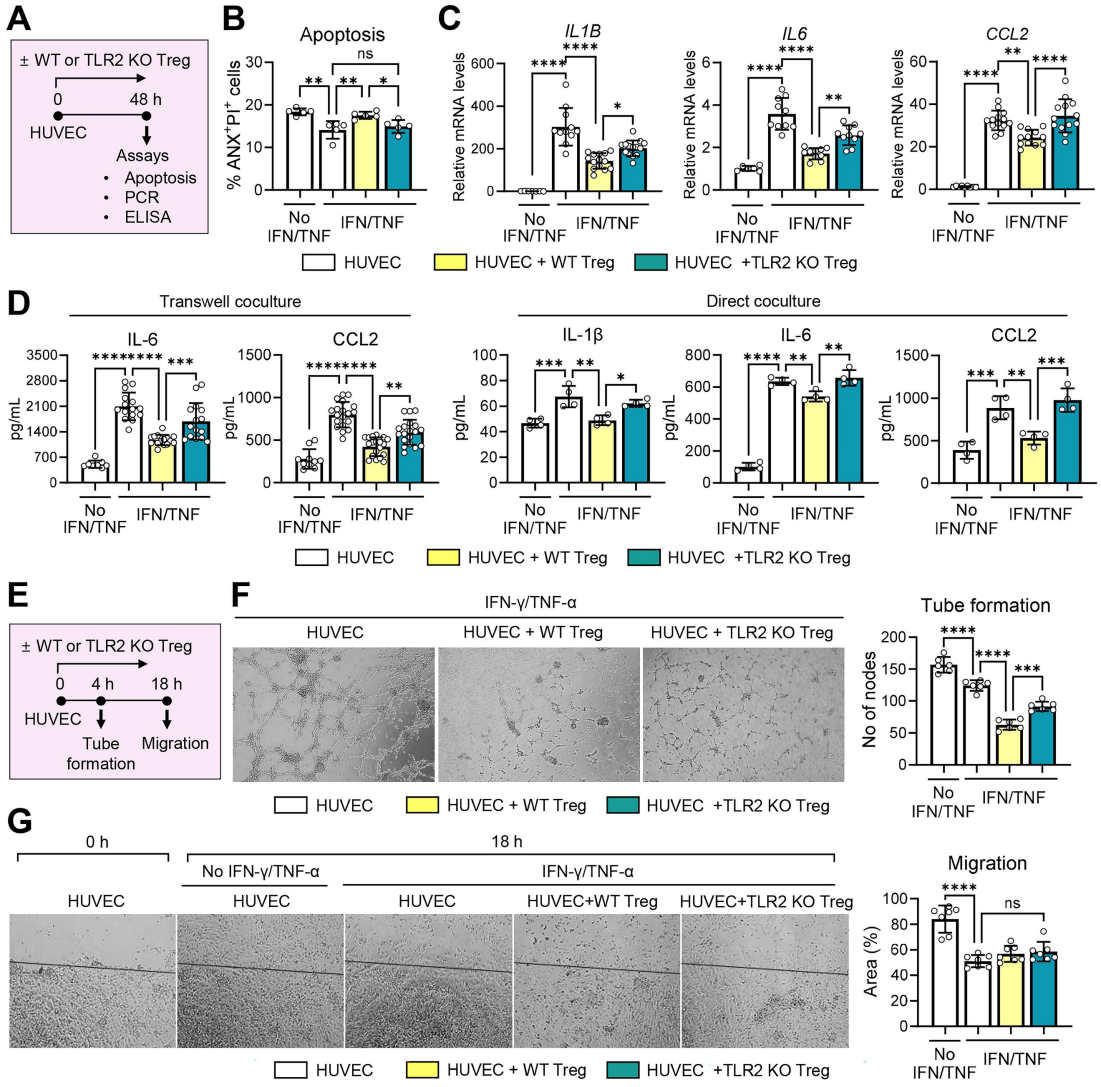
** TLR2 KO Tregs demonstrate reduced suppressive effects on vascular endothelial cells. A.** Experimental scheme for evaluation of effects of WT (C57BL/6) and TLR2 KO Tregs on vascular endothelial cells. CD4^+^CD25^+^Foxp3^+^ cells, isolated from the spleens of WT mice and TLR2 KO mice and treated with anti-CD3 Ab and IL-2, were cocultured with HUVECs in a transwell or direct coculture system. For inflammatory activation, HUVECs were stimulated with IFN-γ and TNF-α. Two days later, HUVECs were assayed for apoptosis (B) and the expression of inflammatory cytokines and chemokines (C, D). **B.** Quantitative flow cytometry results for ANX^+^PI^+^ cells in HUVECs cocultured with WT or TLR2KO Tregs in a transwell system. **C, D.** qRT-PCR and ELISA for evaluation of transcription and secretion of IL-1β, IL-6, and CCL2 in HUVECs cocultured with WT or TLR2KO Tregs in a transwell or direct system. mRNA levels are presented as fold changes relative to the levels in HUVECs that were neither stimulated with IFN-γ and TNF-α nor cocultured with Tregs. **E.** Experimental scheme. HUVECs were cultured along with WT or TLR2 KO Tregs stimulated with anti-CD3 Ab and IL-2 in a transwell system. For inflammatory activation, HUVECs were treated with IFN-γ and TNF-α. Four hours later, HUVECs were evaluated for tube formation. Eighteen hours after a scratch wound was applied, HUVEC migration was measured (G). **F.** Representative photographs of HUVECs in the tube formation assay and quantification of tube formation. The numbers indicate the nodes of vascular tubes in randomly-selected fields at 100x magnification. **G.** Representative photographs of HUVECs in the migration assay and quantification of cell migration. The black line depicts the reference line. The numbers represent the area of cells filling the gaps from the initial scratch 18 h post-scratch. Mean values ± SD are presented. *p < 0.05, **p < 0.01, ***p < 0.001, ****p < 0.0001, ns: not significant, as analyzed by one-way ANOVA with Tukey's test.

To determine whether TLR2 deficiency directly affects the functions of Tregs, we compared the proliferative and secretory capacities of CD4^+^CD25^+^Foxp3^+^ Tregs isolated from the spleens of TLR2 KO and WT mice (**Figure 7**) as well as their effects on vascular endothelial cells (**Figure 8**).

CFSE-labeled CD4^+^CD25^+^Foxp3^+^ Tregs from TLR2 KO mice or WT mice were cultured for 5 d in the presence or absence of stimulation with soluble anti-CD3 Ab and IL-2 (**Figure 7A**). Analysis of CFSE dilution demonstrated that TLR2 KO Tregs exhibited significantly reduced proliferation compared to WT Tregs, both with and without anti-CD3 Ab/IL-2 stimulation (**Figures 7B-C**).

In addition to impaired proliferation, the expression of intrinsic Treg markers CD25 (IL-2Rα) and Foxp3, which are essential for the maintenance of Tregs and their suppressive functions 33,34, rapidly diminished in TLR2 KO Tregs during the 5-day culture period, even in the presence of anti-CD3 Ab and IL-2, whereas WT Tregs maintained high levels of both CD25 (IL-2Rα) and Foxp3 under anti-CD3 Ab/IL-2 treatment (**Figure 7D-E**). Consequently, the percentage of CD4^+^CD25^+^Foxp3^+^ cells was dramatically lower in cultures of TLR2 KO Tregs on day 5 compared to WT Treg cultures (**Figure 7D-E**). Furthermore, the expression of all 3 subunits of the IL-2R complex (IL-2Rα, IL-2Rβ, and IL-2Rγ) was markedly decreased in TLR2 KO Tregs compared to WT Tregs (**Figure 7F**).

Additionally, TLR2 KO Tregs displayed reduced IL-10 transcript levels and lower secretion of IL-10 protein compared to WT Tregs (**Figure 7G**). Conversely, the secretion of IFN-γ protein was significantly higher in TLR2 KO Tregs (**Figure 7G**).

### TLR2 KO Tregs display decreased suppressive activity on vascular endothelial cells

Next, we evaluated the direct effects of TLR2 KO Tregs compared to WT Tregs on vascular endothelial cells. For this purpose, CD4^+^CD25^+^Foxp3^+^ Tregs, freshly isolated from TLR2 KO mice or WT mice and treated with anti-CD3 Ab and IL-2, were cocultured with HUVECs in the presence or absence of IFN-γ and TNF-α. The HUVECs were subsequently assessed for apoptosis and the expression of pro-inflammatory cytokines and chemokines (**Figure 8A-D**) as well as their tube formation and migration potential (**Figure 8E-G**).

IFN-γ/TNF-α stimulation inhibited HUVEC apoptosis as measured by the percentage of ANX^+^PI^+^ cells (**Figure 8B**). Notably, WT Tregs significantly increased apoptosis in IFN-γ/TNF-α-stimulated HUVECs, whereas TLR2 KO Tregs lost this ability (**Figure 8B**). The percentage of ANX^+^PI^+^ cells was significantly higher in HUVECs cocultured with WT Tregs compared to those cocultured with TLR2 KO Tregs (**Figure 8B**).

In addition, IFN-γ/TNF-α markedly stimulated both transcript and secreted levels of IL-1β, IL-6, and CCL2 in HUVECs (**Figure 8C-D**). Both direct and transwell cocultures with WT Tregs significantly downregulated the levels of IL-6, IL-1β, and CCL2 in IFN-γ/TNF-α-stimulated HUVECs (**Figure 8C-D**). Remarkably, these suppressive effects on HUVEC immune activation were significantly abrogated with TLR2 KO Tregs. Both the transcript and secreted levels of IL-6, IL-1β, and CCL2 were higher in HUVECs cocultured with TLR2 KO Tregs compared to those cocultured with WT Tregs (**Figure 8C-D**).

In the tube formation and migration assays, IFN-γ/TNF-α treatment significantly downregulated both the tube formation and migration potentials of HUVECs (**Figure 8E-G**). Transwell coculture with Tregs further suppressed the tube formation of HUVECs. However, this suppressive effect on HUVEC tube formation was significantly reduced with TLR2 KO Tregs compared to WT Tregs (**Figure 8F**). Neither WT nor TLR2 KO Tregs had any effect on HUVEC migration (**Figure 8G**).

Collectively, the present findings indicate that TLR2 deficiency not only inhibits Treg expansion but also impairs Treg functions, including their ability to attenuate the proliferative and inflammatory responses of vascular endothelial cells.

## Discussion

Our data demonstrate that TLR2 signaling is essential for the expansion and function of CD4^+^CD25^+^Foxp3^+^ Tregs upon sterile corneal injury. Injury-induced CD4^+^CD25^+^Foxp3^+^ Tregs mitigate the propagation of inflammation and pathologic angiogenesis in the cornea by reducing pro-angiogenic, pro-inflammatory monocytes and suppressing the inflammatory activation of vascular endothelial cells.

Tregs, generally defined as CD4^+^CD25^+^Foxp3^+^ T cells, are master regulators of immune responses that maintain peripheral immune tolerance and homeostasis. In addition to their classical role as immunosuppressors, emerging evidence demonstrate that Tregs play a crucial role in a wide range of pathophysiological processes, including angiogenesis, to protect tissues from injury and promote repair 35,36. As much as angiogenesis is essential for both normal tissue regeneration and pathological processes, the role of Tregs in angiogenesis is dynamic and highly dependent on tissue and disease contexts, leading to either pro-angiogenic or anti-angiogenic effects 30.

The pro-angiogenic effects of Tregs are well documented, particularly in the context of tumors. Multiple studies have observed a positive correlation between the levels of intratumoral Tregs, VEGF signaling, and tumor angiogenesis in patients 37-40. Additionally, Tregs have been shown to produce pro-angiogenic factors 41,42 or to reduce the numbers of immune cells that inhibit angiogenesis 43,44, thereby enhancing tumor growth. Conversely, the anti-angiogenic effects of Tregs have been identified in states of ischemia or inflammation as well as in tissues characterized by immunologic privilege. For example, Tregs have been associated with a reduction of post-ischemic NV in mouse models of ischemic hindlimb 45 and heart failure 46. Furthermore, the administration of Tregs has been shown to diminish VEGF-driven inflammation or inhibited angiogenesis in mice with skin or airway inflammation 47,48. These anti-angiogenic and anti-inflammatory roles of Tregs have also been demonstrated in the retina and cornea. In a model of oxygen-induced retinopathy, Tregs were found to transiently increase after acute ischemic injury and subsequently decline with time 49. The expansion of Tregs through either an IL-2/anti-IL-2 Ab complex or intravenous administration of Tregs significantly reduced VEGF levels and downregulated the pro-inflammatory activation of microglia, leading to marked attenuation in neovascular retinopathy 49. In the cornea, subconjunctival injection of Tregs inhibited excessive inflammation and potentiated epithelial regeneration after alkali burn injury, thereby alleviating corneal NV and fibrosis 50. Also, it has been reported that peripherally-derived induced Tregs isolated from low-risk corneal transplant recipients effectively suppressed immune rejection of corneal grafts in recipients at high risk of graft rejection 51.

In line with these previous studies, our present findings indicate that adoptive transfer of injury-induced Tregs via either the subconjunctival or IP route significantly reduced monocyte numbers and corneal NV development after suturing injury to the cornea, thus confirming the anti-angiogenic role of Tregs in the cornea. Further mechanistic studies revealed that Tregs exerted direct effects on vascular endothelial cells by increasing apoptosis, suppressing inflammatory activation, and reducing tube formation potential. These findings demonstrate that Tregs restore angiogenic and immunologic homeostasis in the cornea after injury through both their canonical suppressive functions on immune cells and their non-canonical functions on vascular endothelial cells. As elaborated in a recent review 36, it is also possible that Tregs maintain corneal homeostasis through interactions with other tissue-resident non-immune cells, such as corneal epithelial cells and stromal keratocytes, which presents an important area for future exploration.

Another notable observation from our study is that TLR2 deficiency, whether through germline deletion or Ab-mediated neutralization, failed to increase the number of Tregs after corneal injury, correlating with increased levels of pro-angiogenic, pro-inflammatory monocytes and corneal NV. In contrast, the number of Tregs did not differ between TLR2 KO mice and WT mice in the steady state without injury. These findings suggest that TLR2 plays distinct roles in Tregs under homeostatic and pathological conditions and that TLR2 signaling is required for Treg expansion during injury. This observed link between TLR2 and Tregs can be partly explained by the decreased IL-2 levels observed in TLR2 KO mice after injury, as IL-2 is a key cytokine crucial for Foxp3 induction and Treg differentiation both extrathymically and in the thymus 33.

In addition to its effects on Treg numbers, our study revealed that TLR2 deficiency altered the functional quality of Tregs. *In vitro*, TLR2 KO Tregs had markedly lower proliferation compared to WT Tregs, both with and without anti-CD3/IL-2 stimulation. Moreover, TLR2 KO Tregs lost expression of Foxp3 and CD25 (IL-2Rα), which are indispensable for the maintenance of Tregs and their suppressor functions 33,34, more rapidly over time in culture compared to WT Tregs. Furthermore, IL-10 secretion was significantly lower, whereas IFN-γ production was higher, in TLR2 KO Tregs compared to WT Tregs. The ability of Tregs to induce apoptosis in vascular endothelial cells and downregulate their pro-inflammatory activation and tube formation was similarly diminished in TLR2 KO Tregs. This reduction can be explained in part by the decreased IL-10 levels in TLR2 KO Tregs, as IL-10 is known to inhibit proliferation and promote apoptosis of HUVECs 52 as well as attenuate the endothelial inflammatory response 53.

Similar to our findings, previous studies have demonstrated the involvement of TLR2 in the expansion and function of Tregs. Netea et al. reported that CD4^+^CD25^+^ Treg numbers and IL-10 levels were significantly reduced in TLR2 KO mice, but not in TLR4 KO mice, compared to WT littermate controls, and that monocyte recruitment to the site of infection and IFN-γ levels were increased in TLR2 KO mice following infection with *Candida albicans* 54. Similarly, Sutmuller et al. showed that triggering TLR2 with the TLR2 ligand Pam_3_Cys augmented Treg proliferation both *in vitro* and *in vivo*, while TLR4 stimulation with LPS or TLR9 ligation with CpG did not 55. Also, Mazmanian's group demonstrated that PSA of *Bacteroides fragilis* directed the development of inducible Foxp3^+^ Tregs producing IL-10 and enhanced their suppressive capacity in a TLR2-dependent manner 7,8. Collectively, these reports, along with our findings, indicate that TLR2 signaling is essential for both the expansion and sustained functionality of Tregs.

It is important to note that TLR4 deficiency did not affect the numbers of Tregs or monocytes, nor did it influence the development of corneal inflammatory NV after suturing injury in our study. In contrast to our findings, systemic TLR4 inhibition with Eritoran has been demonstrated to decrease the infiltration of CD11b^+^ cells in the cornea and MHC-II^hi^CD11b^+^ cells in draining LNs, subsequently abrogating corneal inflammation in a model of dry eye disease 56. Similarly, TLR4 activation via the topical application of HMGB1 or LPS was shown to enhance the recruitment of endothelial progenitor cells and corneal NV in an alkali-induced corneal injury model, and TLR4 inhibition using a topical HMGB1 antagonist or LPS-RS reversed this phenotype 24. These findings suggest that different TLRs may have distinct roles depending on the specific disease context. Further research is necessary to elucidate the functions of various TLRs, including TLR1 and TLR6 which form heterodimers with TLR2, in the context of corneal diseases, given that a range of TLRs (TLRs 1-10) have been detected in the corneal epithelium 57.

Moreover, CD4^+^CD25^+^ Tregs express higher levels of various TLRs, such as TLR5, TLR7, and TLR8, as well as TLR2 and TLR4, compared to CD4^+^ effector T cells 58,59. Several studies have reported that TLR8 stimulation can reverse the immunosuppressive function of human CD4^+^ Tregs 60,61, whereas TLR5 stimulation can enhance their suppressive function and Foxp3 expression 62. These findings point to the complex interactions between different TLRs and Tregs. Given that TLRs are critical components of the innate immune system that connect innate and adaptive immunity, the specific roles of TLRs in Treg function warrant further investigation.

In conclusion, we demonstrate that TLR2 deficiency exacerbates pathological corneal NV following sterile injury by impairing the expansion and function of CD4^+^CD25^+^Foxp3^+^ Tregs. Our data reveal intricate connections among TLR2, Tregs, monocytes, and vascular endothelial cells in restoring corneal immunologic and angiogenic homeostasis during injury, highlighting the crucial role of CD4^+^CD25^+^Foxp3^+^ Tregs in this process. These findings provide a foundation for the development of Treg-based therapeutic strategies to manage pathological inflammation and NV in the eye and other tissues.

## Supplementary Material

Supplementary figures and tables.

## Abbreviations

Ab: antibody
ANX: Annexin V
BM: bone marrow
CFSE: carboxyfluorescein succinimidyl ester
CLN: cervical lymph node
DAMP: damage-associated molecular pattern
ELISA: enzyme-linked immunosorbent assay
FACS: fluorescence-activated cell sorting
FBS: fetal bovine serum
HBSS: Hank's balanced salt solution
HUVEC: human umbilical vein endothelial cell
IP: intraperitoneal
KO: knockout
LPS: lipopolysaccharide
MACS: magnetic-activated cell sorting
MDSC: myeloid-derived suppressor cell
NV: neovascularization
PAMP: pathogen-associated molecular pattern
PSA: polysaccharide A
PBS: phosphate-buffered saline
PI: propidium iodide
PS: penicillin-streptomycin
qRT-PCR: quantitative reverse transcription polymerase chain reaction
rh: recombinant human
rm: recombinant mouse
TLR: Toll-like receptor
Treg: regulatory T cell
WT: wild-type
