# Supplementary material for: Toll-like receptor 2 deficiency exacerbates corneal angiogenesis in injury by impairing regulatory T cells
Source: Theranostics. 2025 May 7;15(13):6082–99. doi: 10.7150/thno.110322 (PMC12159752; doi:10.7150/thno.110322)
Supplement: Supplementary file 1 — Supplementary figures and tables. [file thnov15p6082s1.pdf]

Supplementary Figures

Figure S1. Comparison of the corneas from WT and TLR2 KO mice in the steady state

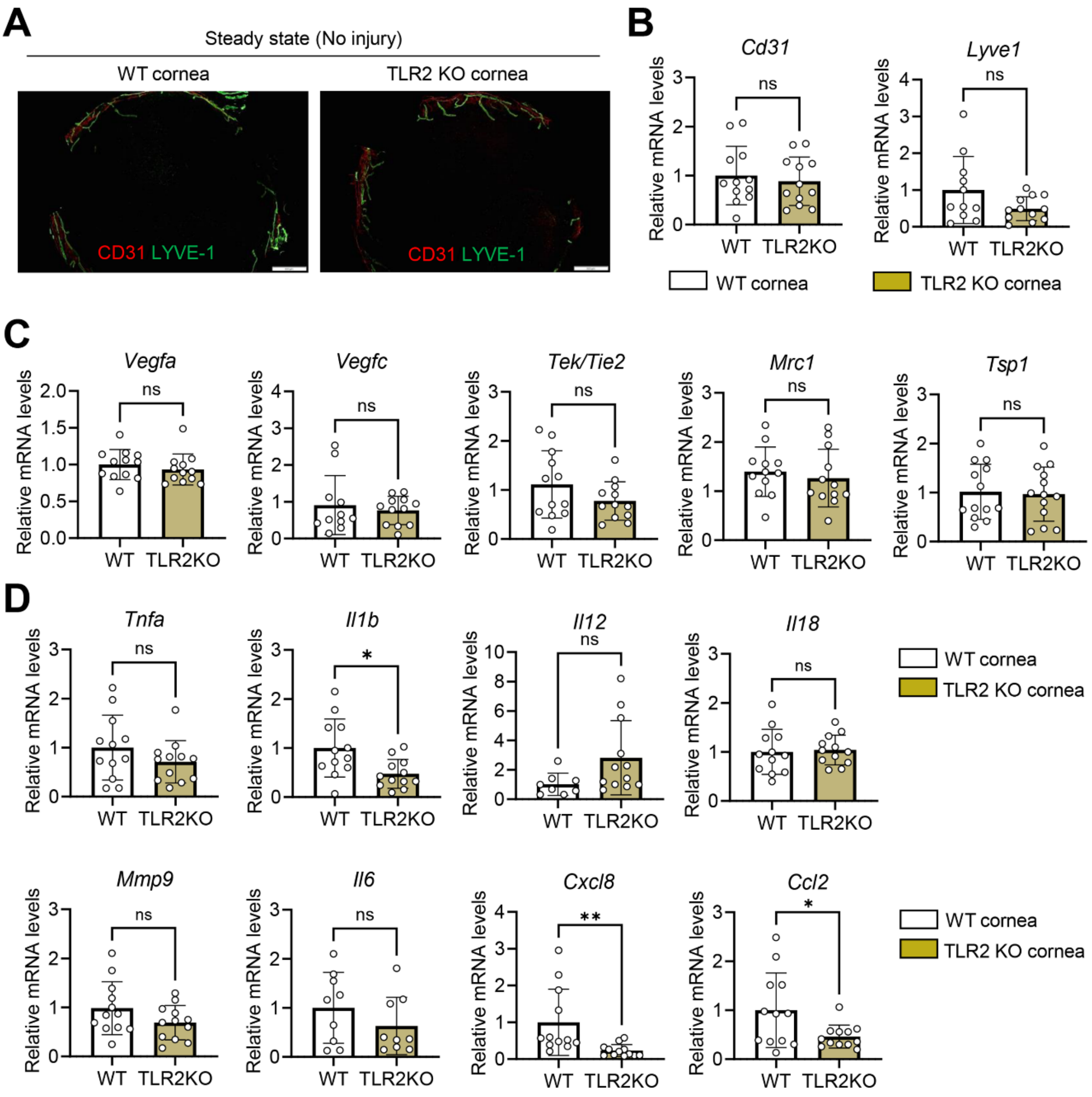

A. Double immunostaining of corneal whole mounts from WT (C57BL/6) mice and TLR2 KO mice with CD31 (red) and LYVE-1 (green)

B. qRT-PCR assays of WT cornea and TLR2 KO cornea for *Cd31* (the pan-endothelial marker) and *Lyve1* (the lymphatic vessel marker)

C. qRT-PCR assays for vascular growth factors (*Vegfa* and *Vegfc*) and pro-angiogenic myeloid cell markers (*Tek/Tie2* and *Mrc1*)

D. qRT-PCR assays for inflammatory cytokines and chemokines

mRNA levels are presented as fold changes relative to the levels in WT corneas. *$p < 0.05$, **$p < 0.01$, ns: not significant, as analyzed by Student's *t*-test

Figure S2. Evaluation of Tregs, monocytes, and granulocytes in TLR4 KO mice and in C57BL/6 mice treated with anti-TLR2 Ab or LPS-RS

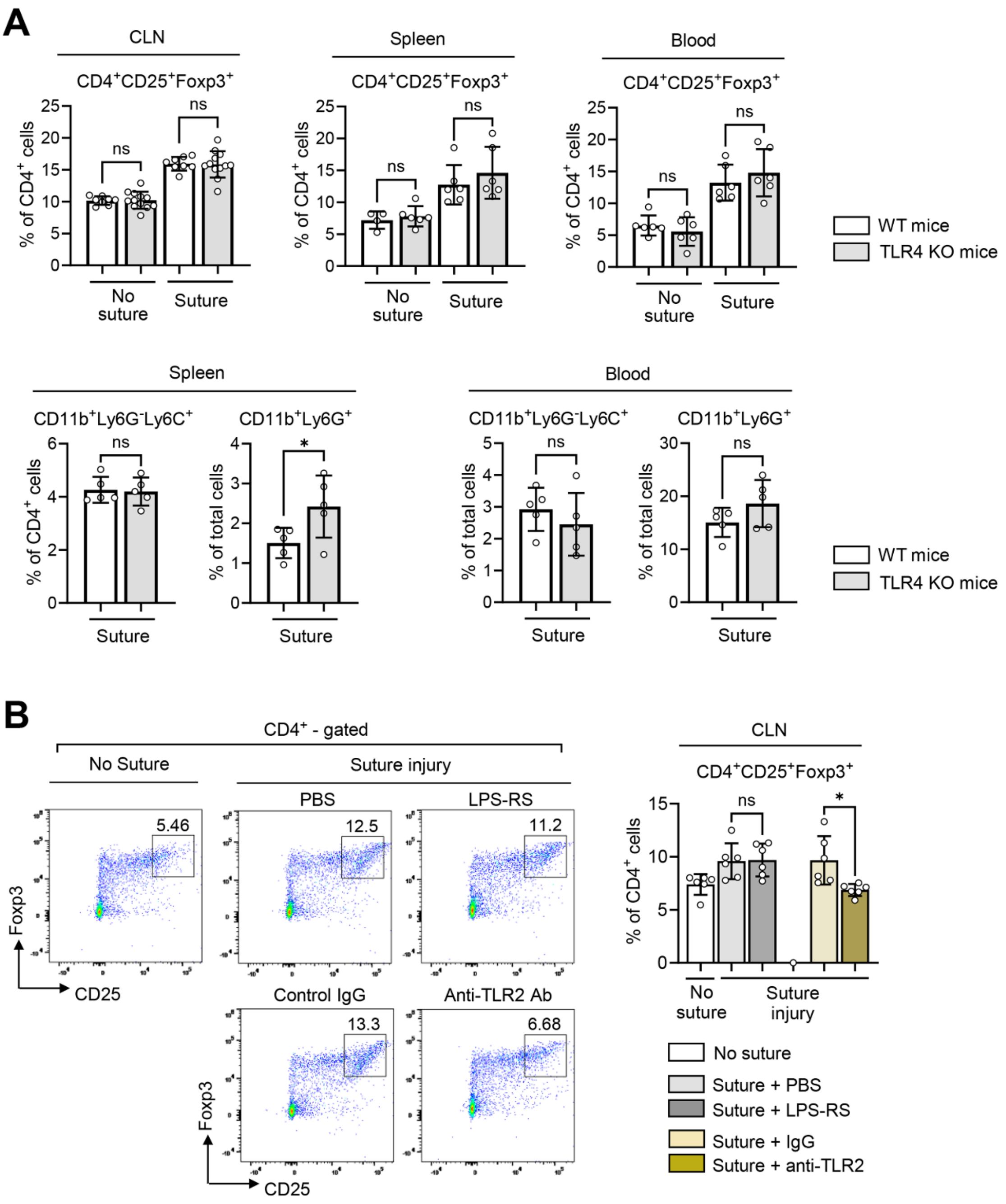

A. Quantitative flow cytometric analysis for $CD4^+CD25^+Foxp3^+$ Tregs, $CD11b^+Ly6G^-Ly6C^+$ monocytes, and $CD11b^+Ly6G^+$ granulocytes in ocular draining cervical lymph nodes (CLN), blood, and spleen of TLR4 KO mice and WT (C57BL/6) mice 7 d after corneal suturing injury

B. Representative and quantitative flow cytometric analysis for $CD4^+CD25^+Foxp3^+$ Tregs in CLN of anti-TLR2 Ab- or control IgG-treated C57BL/6 mice as well as in LPS-RS- or PBS-treated mice 7 d after corneal suturing injury

Mean values ± SD are presented, where each circle represents the data from a single mouse. $^*p < 0.05$, ns: not significant, as analyzed by one-way ANOVA with Tukey's test or by Student's *t*-test

Figure S3. Increased CD11b$^+$Ly6C$^+$ monocytes after injury are pro-inflammatory.

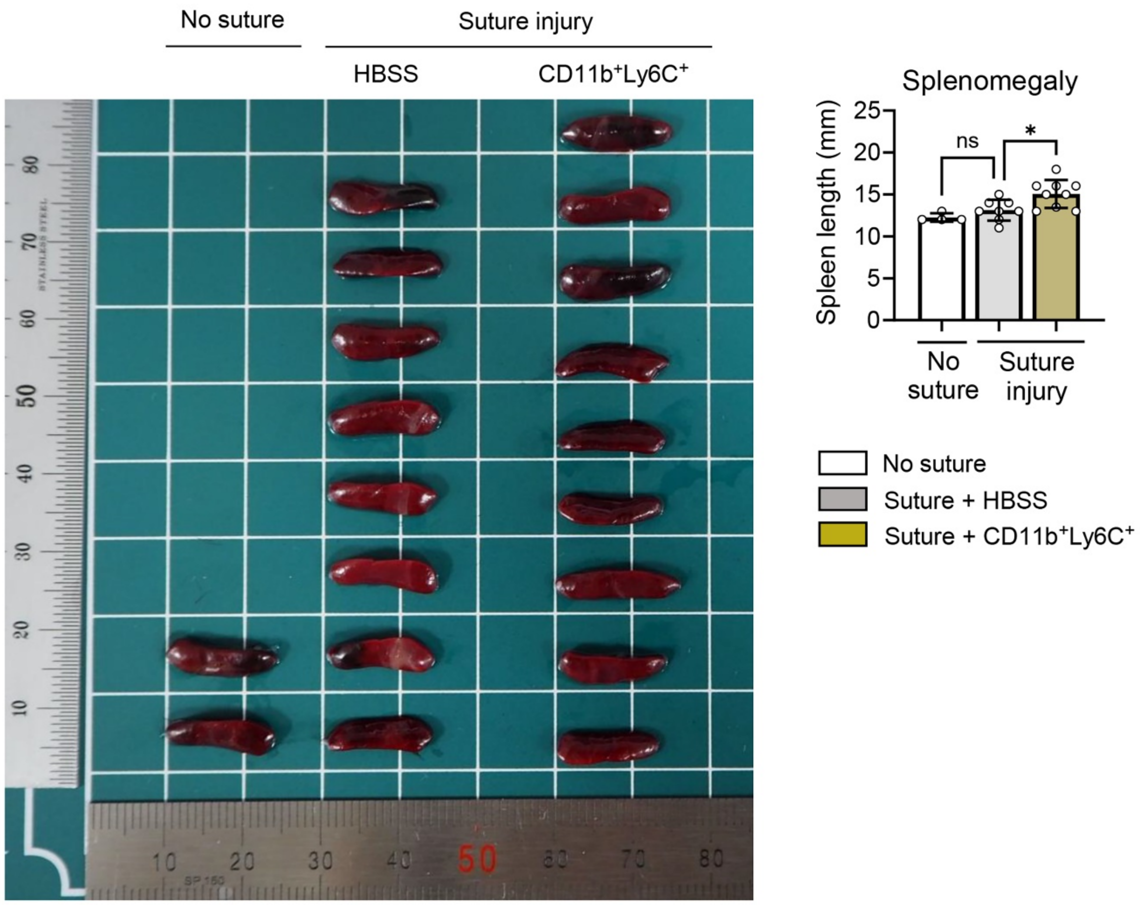

A. Gross photographs of spleen of WT (C57BL/6) mice 7 d after injury and adoptive transfer of CD11b$^+$Ly6C$^+$ monocytes (See Figure 5A–B for experimental scheme)

B. Measurement of splenic size in length (mm)

Mean values ± SD are presented, where each circle represents the data from a single mouse. *$p < 0.05$, ns: not significant, as analyzed by one-way ANOVA with Tukey's test

Figure S4. Sorting efficiency of Tregs from spleen by MACS

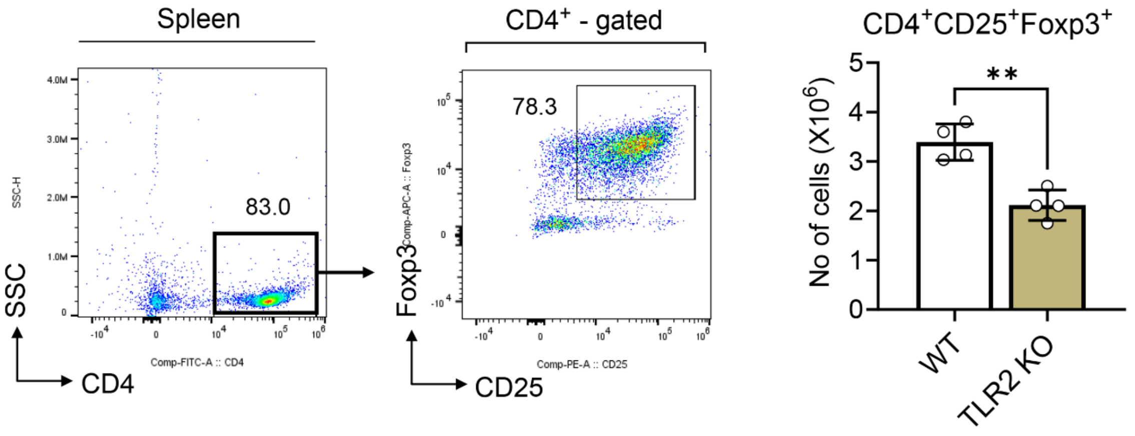

Representative and quantitative flow cytometry data showing isolation of CD4$^+$CD25$^+$Foxp3$^+$ cells from spleens of WT (C57BL/6) mice and TLR2 KO mice using MACS microbeads 7 d after suturing injury to cornea. **$p < 0.01$ as analyzed by Student's t-test
